# Biofilm Localization in the Vertical Wall of Shaking 96-Well Plates

**DOI:** 10.1155/2014/231083

**Published:** 2014-04-13

**Authors:** Luciana C. Gomes, Joana M. R. Moreira, Manuel Simões, Luís F. Melo, Filipe J. Mergulhão

**Affiliations:** LEPABE, Department of Chemical Engineering, Faculty of Engineering, University of Porto, Rua Dr. Roberto Frias, 4200-465 Porto, Portugal

## Abstract

Microtiter plates with 96 wells are being increasingly used for biofilm studies due to their high throughput, low cost, easy handling, and easy application of several analytical methods to evaluate different biofilm parameters. These methods provide bulk information about the biofilm formed in each well but lack in detail, namely, regarding the spatial location of the biofilms. This location can be obtained by microscopy observation using optical and electron microscopes, but these techniques have lower throughput and higher cost and are subjected to equipment availability. This work describes a differential crystal violet (CV) staining method that enabled the determination of the spatial location of *Escherichia coli* biofilms formed in the vertical wall of shaking 96-well plates. It was shown that the biofilms were unevenly distributed on the wall with denser cell accumulation near the air-liquid interface. The results were corroborated by scanning electron microscopy and a correlation was found between biofilm accumulation and the wall shear strain rates determined by computational fluid dynamics. The developed method is quicker and less expensive and has a higher throughput than the existing methods available for spatial location of biofilms in microtiter plates.

## 1. Introduction

Biofilms are defined as structured microbial communities that are attached to a surface and encapsulated within a self-produced matrix [1, 2]. They constitute a serious problem for public health because of the increased resistance of biofilm-associated microorganisms to antimicrobial agents and their potential to cause infections in patients with indwelling medical devices [1, 3].

Intensive studies on the mechanisms of biofilm formation and resistance have encouraged the development of different* in vitro* platforms, such as microtiter plates (MTPs), which are one of the most widely used biofilm model systems [4, 5]. In these systems, biofilms are formed on the bottom and on the wall [6] of the microtiter plate wells (most commonly a 96-well plate) or they are grown on the surface of a coupon placed in the wells of the MTP (most commonly a 6-, 12-, or 24-well plate). The large number of advantages offered by these straightforward and user-friendly systems explain their widespread use (Table 1). In addition, several standard assays are available for the determination of different parameters related to the biofilm in MTPs [7]. They can be categorized into biofilm biomass assays (quantitation of matrix and both living and dead cells), viability assays (determination of viable cells), and matrix quantitation assays (through specific staining of matrix components) (Table 1). Microtiter plates have been intensively used in clinical research for screening of antimicrobial compounds [8, 9] and for studying biofilm formation [10, 11] and inhibition [12, 13]. The most widely used method for following biofilm formation in MTPs is the crystal violet (CV) staining, derived from the original Christensen et al. [14] method, which only measured biofilm biomass at the bottom of the well. CV is a basic dye that stains both living and dead cells by binding to negatively charged surface molecules and polysaccharides in the extracellular matrix of biofilms [15]. Later, the CV assay was modified to increase its accuracy and to allow for biofilm biomass quantitation in the entire well by the solubilization of the dye [16, 17]. This method can therefore be considered a bulk method which provides information about the total amount of biofilms produced without revealing any information about biofilm localization. It has been shown that the biofilm distribution in the wall of a 96-well MTP may not be uniform when dynamic conditions are used (when the MTP is shaken with an orbital motion) [6]. This biofilm heterogeneity can be analyzed, for instance, by microscopy either using standard optical microscopy [18], confocal laser scanning microscopy (CLSM) [19], or scanning electron microscopy (SEM) [20]. The relative advantages and limitations of these two later techniques are presented in Table 2. It is interesting to point out that, for the most part, these microscopy analyses are made at the bottom of the well, disregarding the biofilm that is formed on the vertical wall. Both techniques enable spatial localization of the biofilms, but are inherently low throughput techniques, with a high cost and are subjected to equipment availability. The aim of this work was to develop a low cost and high throughput method that would enable the quantitation of the total amount of biofilm produced inside a well but providing the same information about the spatial location of the formed biofilm. A differential CV staining method was here developed by combining the high throughput features of the usual CV staining but enabling spatial localization of the biofilm without the use of expensive equipment.

## 2. Materials and Methods

### 2.1. Computational Fluid Dynamic (CFD) Simulations

Numerical simulations were made in Ansys Fluent CFD package (version 13.0) as previously described [6]. A cylindrical well (diameter of 6.6 mm and height of 11.7 mm) was built in Design Modeller 13.0 and discretized into a grid of 18,876 hexahedral cells by Meshing 13.0. Simulation was made for a shaking diameter of 50 mm and frequency of 150 rpm. The location of the interface was determined by Ansys Fluent, as well as the magnitude of the shear strain rate. The time averaged shear strain rate was obtained by averaging the steady state shear strain rate of the liquid side during a complete orbit.

### 2.2. Biofilm Formation in 96-Well Microtiter Plates

*Escherichia coli* JM109(DE3) from Promega (USA) was used to form biofilms in 96-well microtiter plates because this strain has shown a good biofilm forming ability in both turbulent [21] and laminar [6] flow conditions. Its Its genotype is* end*A1,* rec*A1,* gyr*A96,* thi*,* hsd*R17 (r_k_^−^, m_k_^+^),* rel*A1,* sup*E44, *λ* ^−^, Δ(*lac-pro*AB), (F′,*tra*D36,* pro*AB, *lac*I^q^ZΔM15), and *λ*(DE3). Cells from an overnight culture were prepared as previously described [6] and biofilms were produced by pipetting 20 *μ*L of these cells into six wells of sterile 96-well flat-bottomed microtiter plates (Orange Scientific, USA) filled with 180 *μ*L of nutrient media. The media used was identical to the one described in [22], except for glucose. A glucose concentration of 1 g L^−1^ was used in this work since it has been demonstrated that this concentration originated the maximum biofilm amount at 24 h in different shaking conditions [6]. Microtiter plates were placed for 24 h at 30°C in an orbital shaking incubator with 50 mm of diameter at 150 rpm (CERTOMAT BS-1, Sartorius AG, Germany).

### 2.3. Biofilm Quantitation by Crystal Violet (CV) Staining

**Figure 1:**
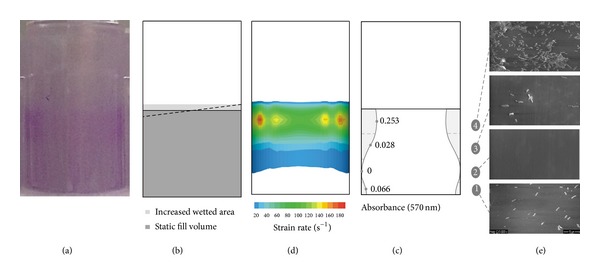
Biofilm localization in shaking 96-well microtiter plates placed in a 50 mm incubator at 150 rpm. (a) Photograph of a well stained with crystal violet; (b) schematic representation of a well where the dark grey area corresponds to the wetted area without shaking, the light grey area represents the area increase upon shaking, and the dotted line depicts the inclination of the air-liquid interface; (c) time averaged shear strain rates (values below 20 s^−1^ are not represented); (d) illustration of the biofilm distribution on the vertical wall assayed by the differential CV staining; (e) representative scanning electron micrographs of the wall sections defined in image (d); (5000x magnification bar = 10 *μ*m).

The amount of biofilm formed was measured using the CV dye in a differential form; this means that the well has been divided into four different sections (Figure 1(d)) and the corresponding vertical wall section was stained sequentially up to a maximum volume of 200 *μ*L. Biofilm quantitation on the bottom of the well was performed by using 25 *μ*L of CV for staining (as detailed below). Section 1 corresponded to a volume between 25 and 50 *μ*L (and a height of 0.77 mm). Section 2 corresponded to a volume between 50 and 100 *μ*L, section 3 corresponded to a volume between 100 and 150 *μ*L and section 4 corresponded to a volume between 150 and 200 *μ*L. Sections 2, 3, and 4 had an equivalent height of 1.54 mm.

Prior to CV staining, the contents of the microtiter plates were discarded and the wells were washed with 200 *μ*L of sterile water to remove non-adherent bacteria [16]. Then, the biofilms were fixed with 250 *μ*L of 96% ethanol [23]. The first section of the well to be quantified was the bottom (not represented in Figure 1(d)), which corresponds to a volume of 25 *μ*L and a height of 0.77 mm. The cells adhered to this region were stained for 5 min with the correspondent volume of 1% (v/v) crystal violet (Merck, Germany) and the dye bound to this specific region was solubilized with 200 *μ*L of 33% (v/v) acetic acid (VWR, Portugal). The absorbance was measured at 570 nm using a microtiter plate reader (SpectraMax M2E, Molecular Devices, UK). The biofilms formed in the successive sections of the well (sections 1 to 4 of Figure 1(d)) were quantified using the described procedure, specifically using the CV volume equivalent to the maximum level of each section (section 1—50 *μ*L; section 2—100 *μ*L; section 3—150 *μ*L; section 4—200 *μ*L). The absorbance corresponding to each of the defined sections and presented on Figure 1(d) was calculated by subtracting the absorbances from the previous sections and considering the dilution factor. To quantify the biofilm formed in each of the well sections, six replica wells were used per experiment and three independent experiments were performed.

### 2.4. Scanning Electron Microscopy (SEM)

Microtiter plate wells containing 24 h old biofilms were imaged by SEM as previously described [24]. Briefly, biofilms were fixed in 3% (w/w) glutaraldehyde in cacodylate buffer, dehydrated with ethanol and hexamethyldisilazane (HMDS), and sputter-coated with a palladium-gold thin film. The different sections along the vertical wall of three independent wells were observed with a SEM/EDS system (FEI Quanta 400FEG ESEM/EDAX Genesis X4 M, FEI Company, USA).

## 3. Results and Discussion

The effect of orbital shaking on biofilm formation in 96-well microtiter plates was firstly assessed by the conventional procedure of crystal violet staining [6, 16]. Through direct observation of the stained wells (prior to solubilization of the dye), it was possible to observe that biofilms were mainly formed on the wall and not on the bottom of the well. Moreover, since the amount of produced biofilms is proportional to the amount of CV adsorbed, it seemed that the biofilm was unevenly distributed in the cylindrical wall and higher amounts were formed closer to the air-liquid interface (Figure 1(a)).

It is widely known that hydrodynamics affects biofilm formation [25–27] as a result of the shear forces that can modulate cell adhesion to a given surface [28–31]. Even though microtiter plates are broadly accepted as biofilm formation reactors by the scientific community, there is still a lack of information on the impact of hydrodynamics on biofilm formation in this system. In this work, the flow inside the wells was simulated using computational fluid dynamics (Figures 1(b) and 1(c)) in order to find out if the hydrodynamic conditions were related to the uneven biofilm distribution that was visible to the naked eye after CV staining (Figure 1(a)). Figure 1(b) represents a scale model of a microwell with the wetted area without orbital motion, the area increase upon shaking, and the inclination of the air-liquid interface. For the shaking conditions chosen for this work (50 mm of orbital diameter and 150 rpm), an area gain of 8.5% and a maximum angle of 7.8° were obtained. Similar to what was observed in the photograph of the stained well (Figure 1(a)), the shear strain rate distribution was not uniform along the wall, being much higher in the liquid side near the interface (Figure 1(c)). In this region, CFD simulations revealed some spots, corresponding to a maximum shear strain rate of 180 s^−1^, which are related to regions of unstable vortices [6].

Observing the results of the differential CV staining (Figure 1(d)), the amount of biofilm detected along the different sections of the wall was not constant. The highest value of absorbance (corresponding to the highest amount of biofilm biomass) was measured in the region located immediately below the liquid level (section 4). In the intermediate sections of the well, the absorbance values were about 10 times lower than near the interface (section 3) or almost zero (section 2), increasing slightly in the section closer to the bottom of the well (section 1).

In order to validate the results obtained by the differential CV staining, SEM analysis was performed on the vertical wall of the wells. Figure 1(e) shows scanning electron micrographs representative of the four sections defined in the application of the differential CV staining (Figure 1(d)). SEM analysis showed that* E. coli* adhesion varied across the wall and that higher attachment occurred closer to the air-liquid interface (section 4) compared to the intermediate (sections 2 and 3) and near bottom (section 1) regions of the well. Biofilms consisting of bacterial aggregates were observed near the interface, while in the lower regions of the well there were only few cells randomly distributed on the surface (Figure 1(e)).

Most likely the spatial location of the biofilms (assayed by differential staining and SEM analysis) is conditioned by the non-homogeneous distribution of shear strain calculated by CFD during shaking. The higher cell density of the biofilms formed closer to the interface is probably due to the higher oxygen and substrate mass transfer from the bulk solution to the biofilm [32], resulting from a more efficient liquid mixing in this region. These results are in agreement with previous studies [29, 33, 34] showing that higher shear forces promote the formation of denser biofilms.

Both methods used for biofilm analysis in this work (differential CV staining and SEM) enabled a higher detail in analysis of biofilms grown in 96-well microtiter plates, which is not achieved by the spectrophotometric methods commonly used in MTPs (Table 1). Indeed, methods such as the traditional CV assay provide bulk data from a biofilm and are classified as macroscale methods, whereas SEM is a microscale technique [24]. It is interesting to notice that the differential CV staining presented in this work corresponds to an intermediate scale between the traditional CV assay and SEM analysis. The traditional CV assay quantifies the biofilm formed on the wall and bottom of each well of a microtiter plate, which corresponds to an area of about 146 mm^2^ (for a 96-well plate), while the method proposed here evaluates wall sections of about 14 and 28 mm^2^. In comparison, the area covered by a SEM image taken at 5000x magnification was of 2 × 10^−3^ mm^2^, and this technique enables detailed analysis of individual cells within the biofilm rather than simply determining their localization. SEM offers higher magnification (ranging from 20x to approximately 30,000x) and resolution (from 50 to 100 nm), together with the ability of imaging complex shapes (Table 2). It is also highly recommended for the visualization of cellular morphology, cell-to-cell interactions, and matrix components within biofilms [35]. However, one must bear in mind that most laboratories are not equipped with an electron microscope and this technique has a considerably lower throughput than the methods described in Table 1.

Observation of biofilms formed on the bottom of the wells of microtiter plates is very common using optical microscopes at magnifications of 100 to 200x, which typically cover areas of 0.3 mm^2^ [36–39]. These observations have provided some information about the architecture of the biofilms formed on the bottom of the wells (particularly when CLSM is used) but usually disregard the biofilm that forms on the vertical wall. It has been shown that in dynamic conditions the amount of biofilm formed on the vertical wall can be higher than the one formed on the bottom of the well [6], and therefore a method was developed in this work to determine the spatial localization of these biofilms in a high throughput manner, using common laboratory equipment. The area under analysis in each of the four sections defined for the differential staining is equivalent to the area analyzed by optical microscopy using a 15x magnification. This area could be further reduced (thus increasing the precision of the method) by dividing the well in sections of smaller height.

The novel approach presented in this work demonstrates that the CV dye can be extremely useful in locating the adherent cells in microtiter plates when used in a differential way. The method is slightly more laborious and slower than the traditional staining procedure, but it requires fewer resources and has higher throughput than other techniques that are used to determine the spatial location of biofilms.

## Figures and Tables

**Table 1:** Advantages of MTPs as biofilm reactors and standard methods for quantitation of biofilm parameters in MTPs.

Advantages	References	Quantitation assays	References
High throughput	[4, 40]	Biofilm biomass	[1, 16]
Small volumes of reagents	[4, 40]	Microbial physiological activity	[7, 41]
Automation	[42, 43]	Microbial cells in the biofilm	[7, 41]
Multiplexing	[4]	Biofilm matrix	[44]

**Table 2:** Advantages and limitations of scanning electron microscopy (SEM) and confocal laser scanning microscopy (CLSM) on biofilm analysis.

Technique	Advantages	Limitations	References
Scanning electron microscopy	High resolution Wide range of magnifications Good comparative information Ability to image complex shapes	Not real time Requires additional sample preparation Limited quantification	[45–49]

Confocal laser scanning microscopy	Living, fully hydrated samples Non-invasive Quantitative evaluation Reflection and fluorescence mode	Low resolution Narrow range of magnifications Not applicable to thick biofilms	[35, 47, 50–52]
